# Epstein–Barr Virus-Associated T- and NK-Cell Lymphoproliferative Diseases: A Review of Clinical and Pathological Features

**DOI:** 10.3390/cancers13133315

**Published:** 2021-07-01

**Authors:** Charlotte Syrykh, Sarah Péricart, Claire Lamaison, Frédéric Escudié, Pierre Brousset, Camille Laurent

**Affiliations:** 1 Department of Pathology, Cancer Institute University of Toulouse-Oncopole, CEDEX 9, 31059 Toulouse, France; syrykh.charlotte@iuct-oncopole.fr (C.S.); pericart.sarah@iuct-oncopole.fr (S.P.); escudie.frederic@iuct-oncopole.fr (F.E.); brousset.p@chu-toulouse.fr (P.B.); 2 Department of Pathology, CHU Pontchaillou, 35000 Rennes, France; claire.lamaison@uni-rennes1.fr; 3 Cancer Research Center of Toulouse (CRCT), INSERM UMR1037, CEDEX 1, 31037 Toulouse, France; 4 ERL 5294 National Center for Scientific Research (CNRS), 31100 Toulouse, France; 5 University of Toulouse III Paul Sabatier, CEDEX 9, 31062 Toulouse, France; 6 Institut Carnot Lymphome CALYM, 69495 Pierre-Bénite, France; 7 Laboratoire d’Excellence ‘TOUCAN’, CEDEX 1, 31037 Toulouse, France

**Keywords:** Epstein–Barr virus, EBV, EBV-associated T/NK-cell lymphoproliferative disorders, chronic active EBV infection, systemic EBV-positive T-cell lymphoma of childhood, EBV-positive aggressive NK-cell leukemia, extra nodal NK/T-cell lymphoma nasal type, primary EBV-positive nodal T/NK-cell lymphoma

## Abstract

**Simple Summary:**

In most Epstein–Barr virus (EBV)-infected individuals, the virus establishes a lifelong latent infection with no specific clinical manifestation. However, EBV primary infection and secondary reactivation may cause various EBV-associated lymphoproliferative disorders (LPD), including hematologic malignancies. Among them, EBV-positive T/NK LPD are uncommon diseases defined by the proliferation of T- or NK-cells infected by EBV, more commonly encountered in Asians and Latin Americans. They encompass a spectrum of disorders ranging from indolent reactive lesions to malignant and aggressive diseases. Despite novel insights from high-throughput molecular studies, the pathogenesis of these disorders is not well understood, and EBV-positive T/NK LPD diagnoses remain challenging due to their rarity and considerable overlap. Indeed, this article discusses new insights into EBV-positive T/NK LPD and focuses on diagnosis challenges, describing the difficulties to clarify the borders between overlapping LPD subtypes.

**Abstract:**

Epstein–Barr virus (EBV) is a ubiquitous virus detected in up to 95% of the general population. Most people are asymptomatic, while some may develop a wide range of EBV-associated lymphoproliferative disorders (LPD). Among them, EBV-positive T/NK LPD are uncommon diseases defined by the proliferation of T- or NK-cells infected by EBV. The 2017 World Health Organization (WHO) classification recognizes the following entities characterized by different outcomes: chronic active EBV infection of T- or NK-cell types (cutaneous and systemic forms), systemic EBV-positive T-cell lymphoma of childhood, EBV-positive aggressive NK-cell leukemia, extra nodal NK/T-cell lymphoma nasal type, and the new provisional entity known as primary EBV-positive nodal T/NK-cell lymphoma. In addition, EBV associated-hemophagocytic lymphohistiocytosis is part of EBV-positive T/NK LPD, but has not been included in the WHO classification due to its reactive nature. Despite novel insights from high-throughput molecular studies, EBV-positive NK/T-cell LPD diagnoses remain challenging, especially because of their rarity and overlap. Until now, an accurate EBV-positive NK/T LPD diagnosis has been based on its clinical presentation and course correlated with its histological features. This review aims to summarize clinical, pathological and molecular features of EBV-positive T/NK LPD subtypes and to provide an overview of new understandings regarding these rare disorders.

## 1. Introduction

Epstein–Barr virus (EBV) is a linear, double-stranded DNA gamma herpes virus which affects more than 90% of the worldwide population [1]. EBV primo-infection occurs through oral transmission in oropharyngeal epithelial cells, where the virus undergoes its first lytic replication and expresses its viral DNA genome, resulting in the production of new viral particles. Then, EBV affects naïve B-cells and establishes latent infection, characterized by the persistence of episomal viral genomes in the nuclei of memory B-cells. These cells can therefore further circulate in peripheral blood and constitute the main persistent EBV reservoir. There are three types of latency programs, based on the expression or co-expression of EBV latency molecular markers, including six nuclear antigens (EBNA1, EBNA2, EBNA3A, EBNA3B, EBNA3C, and EBNA-LP), three latent membrane proteins (LMP1, LMP2a and LMP2B) and two short non-coding RNAs (EBER1 and EBER2). Expression of the full set of EBV-encoded proteins, referred to as type III latency program, is activated in infected naïve B-cells following EBV primo-infection. In order to escape immune surveillance, and to allow infected cells to survive, there is a restriction in the latent gene transcription machinery with a lack of EBNA2 expression (type II latency). Finally, the type I latency program is limited to the expression of EBNA1 and EBERs and is involved in the maintenance and replication of the episomal viral genome [2]. EBV latent antigens are able to impact both the growth and transformation of B-cells, which may lead to the development of subsequent lymphomas. Cells with restricted EBV gene expression (latency I and II) present no autonomous growth potential, thus malignant transformation of such cells requires additional factors (genetic/cytogenetic changes, cytokines or cellular interactions) [2]. Two genetically different EBV forms (EBV type 1 and 2) are distinguished by allelic polymorphisms in the latent-cycle genes encoding the nuclear antigens 2 and 3 (EBNA2 and EBNA3, respectively). Both types are detected worldwide, type 1 being the most prevalent, except in central Africa, Papua New Guinea and Alaska, where type 2 is more common [3].

Clinically, EBV primo-infection is usually silent, but symptomatic forms may arise in children and young adults, leading to infectious mononucleosis, where B lymphocytes are highly infected. In most EBV-infected individuals, the virus establishes lifelong latent infection with no specific clinical manifestation. However, EBV primary infections, as well as secondary reactivations, may cause various EBV-associated lymphoproliferative disorders (LPD) including hematologic malignancies, especially within immunocompromised hosts. While most of EBV-associated LPD involve B-cells, EBV-associated T/NK LPD are also reported, mostly appearing in Asia and Latin America and representing a spectrum of reactive, indolent and malignant disorders [4].

The mechanism that allows EBV to infect B-cells through CD21 and HLA class II receptors is well known [5], whereas the infectious process of T/NK-cells is less established [6]. A few years ago, it was hypothesized that NK-cells could hijack CD21 molecules via synaptic transfer from EBV-infected B-cells [7]. It was also suggested that CD21 may be expressed on lymphocyte common progenitors or immature T-cells [8]. Finally, the impairment of balance between the host immune response and EBV replication may lead to various EBV-positive LPD involving B-, T- or NK-cells.

Herein, we discuss the new insights into EBV-positive T/NK LPD, focusing on diagnosis challenges and describing the difficulties to clarify the borders between overlapping LPD subtypes.

## 2. EBV-Positive Hemophagocytic Lymphohistiocytosis

Hemophagocytic lymphohistiocytosis (HLH) is a fulminant life-threatening inflammatory disease due to uncontrolled hyperactivation of the immune system. Its diagnosis is based on the presence of 5 out of 8 of the following HLH criteria: fever, splenomegaly, cytopenias, hypertriglyceridemia or hypofribrinogenemia, hemophagocytosis, low or absent NK-cell activity, hyperferritinemia, and high soluble CD25 levels [9]. However, these symptoms and biological abnormalities are not specific to HLH, and some of them may occur during any trivial primary EBV infection. Timing and severity of these signs help make the distinction.

Two types of HLH are recognized: a primary (i.e., familial) form related to various genetic alterations, and a secondary one [10,11,12]. Most of the genetic alterations described in primary HLH impair NK- or T-cell functions—*PRF1*, *UNC13D*, *STX11*, *STXBP2*, and *SH2D1A* mutations causing familial HLH2, HLH3, HLH4, HLH5, and X-linked lymphoproliferative disease type 1 (XLP1), respectively. On the contrary, other less common primary HLH such as the X-linked lymphoproliferative disease type 2 (XLP2) present normal cytotoxicity and are due to dysregulated inflammasome or TNFR responses [13,14]. In the absence of any genetic predisposition, secondary HLH is commonly triggered by infections, malignancies or autoimmune disorders, EBV being the most common infection associated with it [10,12,15].

**Figure 1:**
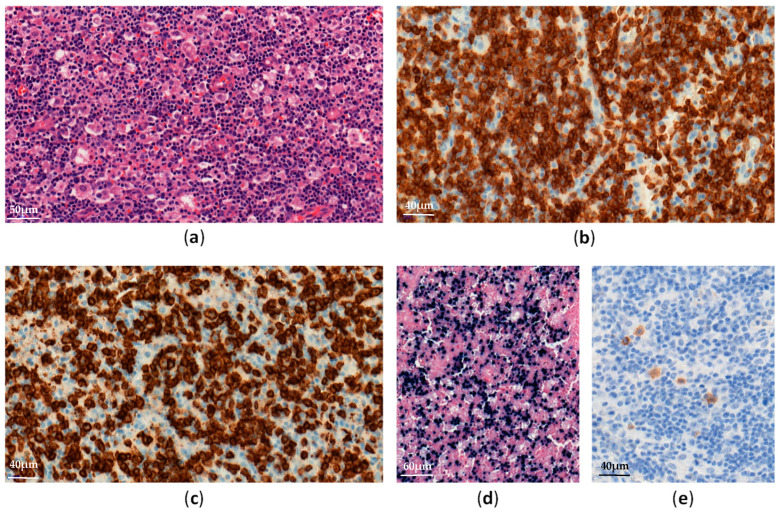
EBV-positive hemophagocytic lymphohistiocytosis in a lymph node: (**a**) Diffuse lymphohistiocytic infiltrate with hemophagocytosis (H&E ×300); (**b**–**e**) Atypical lymphoid cells are positive for CD3 (**b**, ×400), CD8 (**c**, ×400), EBV-encoded small RNA (EBER) (**d**, ×200) and LMP1 (**e**, ×400).

EBV–positive HLH is rare and mainly affects children and adolescents in Asia [16]. Histological analyses show lymphohistiocytic infiltrates with hemophagocytosis and EBV-infected cells, which are small and show slight or no atypia (Figure 1).

EBV-infected cells can be observed in many organs (e.g., lymph nodes, liver, spleen, bone marrow). These cells often have a cytotoxic CD8+ T-cell origin and less frequently an NK-cell phenotype [17]. TCR monoclonal rearrangements may be found in T-cell HLH, due to clonal proliferation of EBV-infected T-cells, but do not correlate with clinical outcomes [18].

Survival for patients with EBV-associated HLH has greatly improved when treated promptly in accordance with the HLH-2004 protocol [9]. However, some patients’ conditions evolve into other EBV-positive T/NK LPD requiring more intensive therapies. In addition, EBV-HLH may also occur at the same time as systemic EBV-positive T/NK-cell lymphoma of childhood.

## 3. Chronic Active EBV Infection, Systemic Form

Systemic chronic active EBV infection (CAEBV) is defined as a chronic systemic illness related to EBV infection. It is characterized by persistent clinical symptoms for more than 3 months, including fever, hepatosplenomagly and lymphadenopathy, without any evidence of underlying immunodeficiency [19]. Most cases have been reported in Asia and Latin America, involving predominantly T- or NK-cells, but EBV-infected B-cells have also been reported in CAEBV patients from the USA [20].

**Figure 2:**
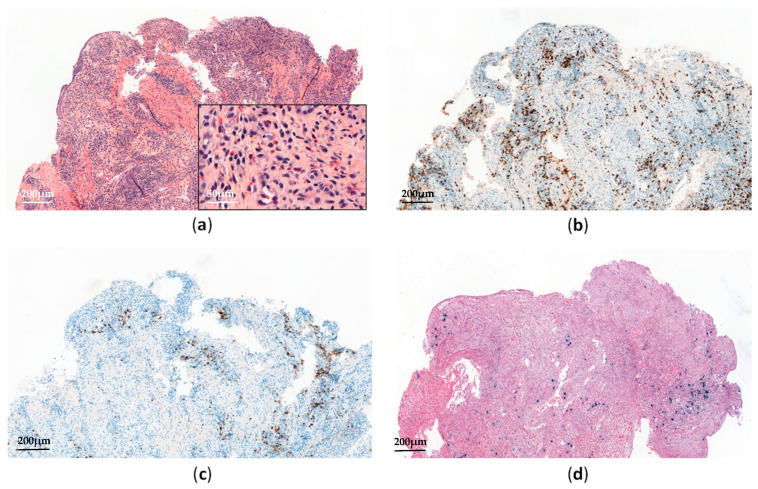
Systemic chronic active EBV infection involving the gastrointestinal tract: (**a**) Esophagus biopsy shows a polymorphic inflammatory infiltrate with a predominance of non-atypical small lymphocytes (H&E, ×60 and ×300); (**b**) The lymphoid infiltrate contains a predominance of CD3+ T-cells (×60); (**c**) Anti-CD20 immunostaining shows scattered reactive B-cells (×60); (**d**) EBV-encoded small RNA (EBER) in situ hybridization shows scattered positive cells (×60).

Histopathological analyses of affected tissues are not specific to CAEBV and usually show non-destructive reactive inflammatory lesions [19]. The diagnosis is made through the detection of elevated EBV DNA levels in peripheral blood and EBV-positive lymphocytes in tissues, showing a type II latency pattern (Figure 2). Monitoring EBV-DNA loads in peripheral blood is also useful for assessing the treatment response and appears to have prognostic value [21].

In Asia, most CAEBV are of T-cell type (60%), being mostly CD4+ rather than CD8+ [4,22]. However, some double positivity (CD4+ and CD8+) and γδ phenotypes have also been reported [20]. Chromosomal aberrations, increasing during the clinical course, were detected in some cases, and monoclonal EBV-infected T-cells have been reported in 50% of patients [4]. Although no genetic defects have been identified yet, some CAEBV families and racial susceptibilities have been described, suggesting genetic predispositions to EBV-mediated immune dysregulations [23,24]. In fact, HLA 26 and 52 loci—frequently seen in Asia and Mexico, respectively—have recently been reported with a higher risk of EBV-positive T/NK LPD [25]. The CAEBV clinical course ranges from indolent presentations (with episodic symptoms and asymptomatic periods) to fulminant presentations leading to death in a few weeks, in the case of no therapeutic intervention [25]. Patients with T-cell type CAEBV present worse outcomes than those with NK-cell type [22,26]. However, some NK-cell CAEBV may also evolve into aggressive NK-cell leukemia (ANKL) or extra-nodal NK/T-cell lymphoma, nasal type (ENKTL) [26]. In a comprehensive review of CAEBV, Arai described recent evidence suggesting the involvement of NF-κB and JAK/STAT pathways in the development of both inflammatory and neoplastic CAEBV aspects [27]. Moreover, a recent whole-exome sequencing analysis performed on 83 CAEBV patients has identified recurrent somatic mutations involving *DDX3X*, *KMT2D*, *BCOR/BCORL1*, *KDM6A*, and *TET2* genes, with at least one somatic mutation detected in 58% of cases [28]. Interestingly, *DDX3X* mutations—known to be associated with hematological malignancies such as Burkitt lymphoma and ENKTL [29,30]—have been reported in serial lymphomas arising from previous CAEBV [25]. This suggests that in such cases, the acquisition of *DDX3X* mutations among others may trigger lymphomagenesis. More recently, Okuno et al. have also reported that EBV genomes in CAEBV patients harbored frequent intragenic deletions that frequently occurred in the *BamHI* rightward transcript microRNA clusters and several genes required for producing viral particles [31]. Such deletions are expected to reactivate the lytic cycle (by upregulating the expression of two immediate early genes, namely *BZLF1* and *BRLF*) and to preclude viral production and cell lysis [31]. Finally, in a recent review article on CAEBV, the authors detailed the potential role of host genetic factors in the pathogenesis of CAEBV. While CAEBV develops in immunocompetent hosts by definition, some patients appear to have minor defects in cellular immunity that may impair immunosurveillance on EBV-infected T/NK-cells [32,33,34].

According to the clinical course and histological data, Ohshima et al. have proposed a classification of systemic CAEBV forms as follows: Category A1 (polyclonal and polymorphic LPD), Category A2 (monoclonal and polymorphic LPD), and Category A3 (monoclonal and monomorphic LPD) [35]. The fourth one, called Category B (monoclonal and monomorphic LPD with a fulminant course), has been considered to be a systemic EBV-positive T-cell lymphoma of childhood in the 2017 World Health Organization (WHO) classification (see below) [19]. Importantly, monoclonality in the proliferation predicts no increase in the mortality rate and should not be diagnosed as a lymphoma [26,35].

## 4. Chronic Active EBV Infection, Cutaneous Form

### 4.1. Hydroavacciniforme-Like Lymphoproliferative Disorder

Hydroavacciniforme-like lymphoproliferative disorder (HV-LPD) is a rare cutaneous form of CAEBV characterized by blistering photodermatoses with vacciniform scars [36,37,38]. It mostly affects children in Asia and Latin America, but adult and elderly cases have also been reported [38,39]. HV-LPD has a broad range of clinical presentations ranging from indolent cutaneous forms to the severe systemic form [19,38]. Classic HV-LPD exhibits lesions typically limited to photoexposed skin, displaying favorable outcomes as it spontaneously regresses in adulthood. However, severe forms of HV-LPD also exist, characterized by extensive and ulcerative skin lesions with systemic symptoms [4,37,38]. Such HV-LPD may eventually evolve into other EBV-positive T/NK LPD, such as systemic CAEBV or systemic EBV-positive T-cell lymphoma, ENKTL, or ANKL [38]. Recent data has suggested that white patients with HV-LPD are less likely to progress into systemic diseases and show a much better prognosis than non-white patients [40]. Skin biopsies show inflammatory infiltrates with necrosis, ulceration, and angiocentricity. EBV-infected cells with type II latency are found in variable proportions and are small or medium-sized cells with cytotoxic CD8+ T-cell (70%) rather than NK-cell phenotypes [37,38].

HV-LPD pathogenesis remains unclear, but like in other EBV-positive T/NK LPD, geographic and racial distributions suggest some genetic predispositions. TCR gene monoclonal rearrangements are found in almost all T-cell type HV-LPD, with no prognosis impact [37,41]. Genomic explorations of HV-LPD have rarely been carried out. A recent whole-exome sequencing analysis performed in five Chinese patients with HV-LPD found five potentially driver mutations involving *STAT3*, *IKBKB*, *ELF3*, *CHD7*, and *KMT2D* genes [42], but these findings need to be confirmed for larger cohorts.

### 4.2. Severe Mosquito Bite Allergy

Severe mosquito bite allergy (SMBA) is another cutaneous manifestation of CAEBV, mostly reported in Asia and Mexico [4,43]. SMBA mainly affects children and adolescents and is characterized by localized skin lesions (erythema, bullae, and ulceration) with variable systemic symptoms (fever, lymphadenopathy, and liver dysfunction) appearing some hours after a mosquito bite. Patients present increased serum IgE titers and high blood EBV DNA loads with NK-cell lymphocytosis [19].

Histologically, skin biopsies show similar lesions to HV-LPD, with more extensive local necrosis and angiodestruction associated with a polymorphic infiltrate rich in eosinophils and histiocytes, admixed with small lymphocytes and larger atypical cells. EBV-infected cells (type II latency) are found in fewer proportions than in HV-LPD and usually have an NK-cell phenotype (CD3ε+, CD56+) [19]. Chromosomal alterations have rarely been identified [4], but full molecular studies on SMBA are lacking in the literature. Finally, SMBA may worsen due to HLH or evolve into other EBV-positive T/NK LPD, such as HV-LPD, systemic CAEBV, or even NK/T-cell lymphoma or ANKL [4].

## 5. Systemic EBV-Positive T-Cell Lymphoma of Childhood

Systemic EBV-positive T-cell lymphoma of childhood (STCLC) is a fulminant systemic T-cell lymphoma that usually occurs in Asian children and young adults, shortly after EBV primo-infection or sometimes following systemic CAEBV (especially monoclonal ones) [19,24,44]. STCLC patients present systemic symptoms with HLH almost always rapidly triggering multi-organ failure and death. Biopsies of affected tissues show proliferation of bland small or medium-sized EBV+ T-cells, sometimes admixed with large and atypical cells [19]. Primary STCLC usually have a cytotoxic CD8+ phenotype, while STCLC that happens after CAEBV are mostly CD4+ [44]. The EBV latency program has not been clearly established yet, and LMP1 is usually negative by immunohistochemistry.

Most of the cases show monoclonal TCR rearrangements [19,45]. Moreover, chromosomal aberrations have been reported in some cases and turned out to be associated with the poorest outcomes [46]. As STCLC have many clinicopatholgical features overlapping with EBV-associated HLH, the identification of karyotypic abnormalities should therefore be helpful to distinguish these two entities. In a recent retrospective study of targeted NGS in a cohort of 169 EBV T/NK-LPD, including 34 STCLC, mutations were detected in 88.2% of STCLC cases, with *KMT2D* being the most frequently mutated gene (17.6%), followed by *MFHAS1* (14.7%), *STAT3* (14.7%), *EP300* (11.8%), *ITPKB* (8.8%), *DDX3X* (8.8%), *NOTCH1* (8.8%), and *NOTCH2* (8.8%) [47].

## 6. Extra-Nodal NK/T-Cell Lymphoma, Nasal Type

ENKTL is an aggressive lymphoma arising in middle-aged adults mostly from Asia and Latin America [48]. ENKTL involves extra-nodal sites, especially in the upper aerodigestive tract, but can also affect skin, soft tissues, the gastrointestinal tract, the central nervous system and lungs [49,50]. During the initial stages, ENKTL patients present localized symptoms such as nasal obstruction or epistaxis. Then, the disease spreads to multiple sites and causes systemic symptoms such as fever, weight loss, and sometimes HLH. Secondary dissemination to lymph nodes may also occur, but the bone marrow is infrequently involved [48,51,52,53].

**Figure 3:**
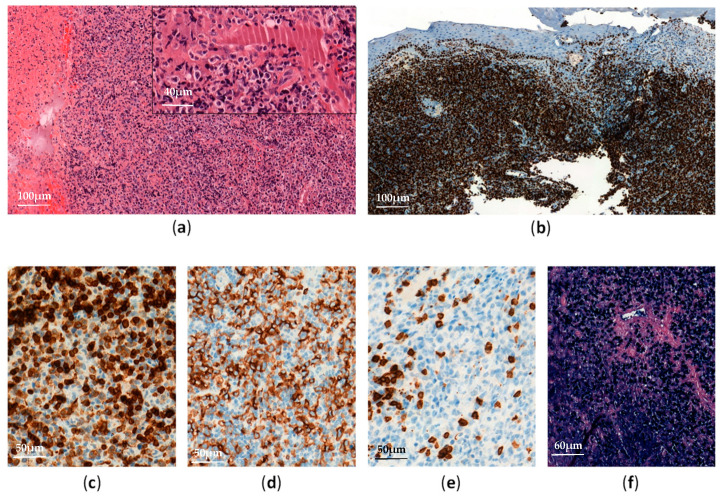
Extranodal NK/T-cell lymphoma, nasal type: (**a**) Nasal mucosa biopsy shows diffuse and expansive lymphoid infiltrate with necrosis (H&E, ×100 and ×400); (**b**) Ki67 immunostaining shows a high proliferative index (90%) of the atypical lymphoid infiltrate (×100); (**c**–**e**) The neoplastic cells are positive for CD3 (**c**, ×300) and CD56 (**d**, ×300), and negative for CD5 (**e**, ×300); (**f**) EBV-encoded small RNA (EBER) in situ hybridization is positive in almost all neoplastic lymphoid cells (×200).

Biopsies of affected tissues show proliferation of medium-sized or large atypical cells with angiocentricity, angiodestruction, and necrosis (Figure 3).

Proliferating cells typically have an NK-cell phenotype—positivity of CD56, CD3-epsilon, and CD2, variable expression of FAS, FASL, CD25, CD38, and CD30, but no expression of surface CD3, CD4, and CD5 [48,54,55]. However, some ENKTL cases have a cytotoxic CD8+ T-cell phenotype with T-cell marker expression (CD3, TCRαβ, or TCRγδ) and monoclonal TCR rearrangements [55,56,57,58]. Importantly, EBV is observed in almost all tumor cells with a frequent type II latency pattern, suggesting that the virus is involved in the early stages of ENKTL lymphomagenesis [48]. Moreover, elevated EBV DNA loads are correlated with poor prognosis [53,59].

**Figure 4:**
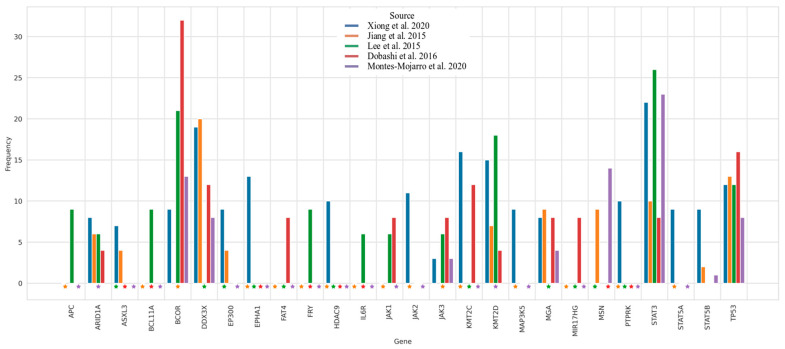
Representation of the genomic alteration frequencies of ENKTL according to the literature. Dataset comes from published data from Xiong et al. Cancer Cell 2020 (*n* = 63 WES and 37 WGS), Jiang et al. Nature Genetics 2015 (*n* = 25 WES and 80 targeted sequencing), Lee et al. Oncotarget 2015 (*n* = 13 WES and 21 targeted sequencing), Dobashi et al. Genes Chromosomes Cancer 2016 (*n* = 25 targeted sequencing), Montes-Mojarro et al. Modern Pathology 2020 (71 targeted sequencing). The top 10 most altered genes contained in each publication have been selected. Stars indicate missing genes in targeted panels.

EBV contribution to the development of ENKTL is not fully understood, but it has been suggested that LMP1, through NFKB signaling activation and microRNAs deregulation, may affect pro- and anti-apoptotic signaling of NK-cells [60,61]. Indeed, in vitro studies showed that LMP1 increases NK-cell sensitivity to IL2, and therefore promotes NK-cell proliferation [62]. Several studies have also reported upregulation of PD-ligand 1 (PDL1) in ENKTL, induced by the release of anti-inflammatory cytokines, oncogenic activation of STAT3 pathway, and LMP1 overexpression. PDL1 overexpression in ENKTL may thus contribute to tumors escaping from immune surveillance and participate in NK-cell growth. Over the years, ENKTL genomics has been part of an active field of research and generated new insights into disease pathogenesis. High-throughput molecular and genomic profiling studies have thus identified abnormalities in genes involved in the apoptotic pathway, such as mutations in *TP53* (20–60%) and *FAS* (50%) [63], as well as various cytogenetic aberrations. The most common one is 6q16-27 deletion, resulting in a loss of key tumor suppressor genes (*PRDM1*, *ATG5*, *AIM1*, *HACE1*, and *FOXO3*) [64]. Other recurrent aberrations include 1q21–q44, 2q and 7q gains, and 17p15–22 loss [64]. In addition, gene expression profiling (GEP) studies have shown deregulations in several oncogenic pathways (cell cycle/apoptosis, NFKB, NOTCH, and JAK/STAT signaling), as well as single gene deregulations (*MYC*, *RUNX3*, and *EZH2*) [65,66,67]. More recently, activating mutations in *BCOR, STAT3*, *STAT5B*, *JAK3*, *DDX3X*, and alterations in epigenetic regulators (*KMT2D*, *KMT2C*, *ASXL3*, *ARID1A*, and *EP300*) have also been detected (Figure 4) [29,68,69,70,71,72].

A recent study showed that *STAT3*, *BCOR*, and *DDX3X* mutations were mutually exclusive, suggesting different molecular pathways involved in ENKTL pathogenesis [72]. Moreover, it should be noted that ENKTL have shown different molecular patterns depending on whether it was developed in Asian or Latin American patients. Indeed, more frequent *TP53*, *BCOR*, and *DDX3X* mutations and fewer *STAT3* ones have been described in Asian patients than in Latin Americans [72]. Finally, genome-wide studies have found *HLA-DPB1* to be associated with an increased risk of developing ENKTL [73].

ENKTL prognosis has improved in recent years, owing to more intensive therapies. Patients at localized stages are usually treated with chemo-radiotherapy only, while L-asparginase plus chemotherapy followed by hematopoietic stem cell transplantation are implemented for advanced stages. Promising new therapies should include anti-PD1/PDL1 and anti-CD38, JAK/STAT and EZH2 inhibitors [60].

## 7. Aggressive NK-Cell Leukemia

**Figure 5:**
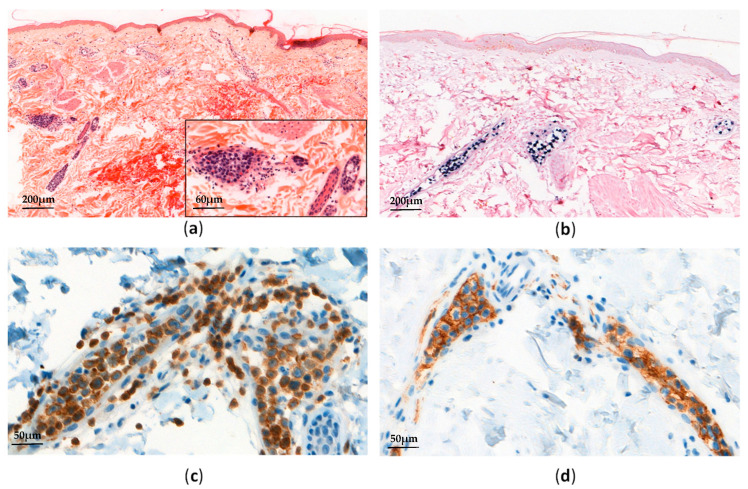
Aggressive NK-cell leukemia: (**a**) Skin biopsy shows an intravascular atypical lymphoid infiltrate (H&E, ×50 and ×200); (**b**) EBV-encoded small RNA (EBER) in situ hybridization is positive in almost all neoplastic lymphoid cells; (**c**,**d**) Neoplastic lymphoid cells are positive for CD3 (**c**, ×300) and CD56 (**d**, ×300).

ANKL is a very rare and malignant LPD of NK-cell type, being closely related to EBV (i.e., 90% of cases). ANKL is mostly found in young or middle-aged adults in Asia, either developing on its own or in the setting of other NK-cell type LPD (i.e., CAEBV or ENKTL) [4,48,74,75]. ANKL shares some clinical manifestations with STCLC (such as systemic symptoms, HLH, or coagulopathy), showing a fulminant course that leads to multi-organ failure. Nevertheless, contrary to STCLC, ANKL patients usually present leukemic EBV-positive NK-cells in their blood. These leukemic NK-cells show a morphological range from normal large granular lymphocytes to atypical lymphocytes with azurophilic granules. In tissues, the infiltration of bone marrow, skin, lymph nodes, the liver, and the spleen can be subtle (Figure 5).

FACS analyses show a mature NK phenotype (CD3ε+, CD56+, and negative for surface CD3, CD5, CD4, and CD8), CD16 and FASL being frequently positive and CD57 negative [75].

ANKL genetic alterations have been documented less often than ENKTL ones, but comparisons between these two entities have revealed some significant differences. Actually, frequent chromosomal aberrations (such as gains of 1q23.1-24.2 and 1q31.3, or losses of 7p15.1–p22.3 and 17p13.1) have been more frequently reported in ANKL than in ENKTL, while a loss of 6q16.1–q27 has been commonly identified in ENKTL and less so in ANKL [64]. More recently, NGS analyses on limited cases have shown recurrent mutations in the JAK/STAT pathway, particularly in *STAT3* and *STAT5B* (ranging from 20% to 48% of cases). Contrary to ENKTL, *JAK3* mutations have not been detected so far in ANKL. Additional mutations affecting RAS-MAPK pathways, *TP53*, *DDX3X* and epigenetic regulators such as *BCOR*, *TET2*, *CREBBP*, and *MLL2* have also been identified, but still require confirmation [47,76,77].

The introduction of l-asparginase and allogenic hematopoietic cell transplantation has improved ANKL therapeutic complete responses. However, its prognosis is extremely poor, with a median survival of less than 1 year [19,75].

## 8. Primary EBV-Positive Nodal T-Cell or NK-Cell Lymphoma

Primary EBV-positive nodal T-cell or NK-cell lymphoma (nodal TNKL) has been recognized as a provisional entity within peripheral T-cell lymphomas, not otherwise specified (PTCL NOS) in the current 2017 WHO classification [48]. This entity that more commonly affects the elderly encompasses EBV-positive PTCL NOS with T-cell or NK-cell phenotypes, primarily involving lymph nodes with no infiltration of nasal or extra-nodal sites [48,78]. The clinical course is usually aggressive.

Histologically, lymph nodes show proliferation of monomorphic (or less frequently, pleomorphic) medium-sized or large cells, sometimes displaying centroblastic morphology, associated with infrequent necrosis or angiodestructive pattern. Thirty to fifty percent of cells are EBV-infected (type II latency) and mostly exhibit a cytotoxic CD8+ T-cell phenotype, frequently showing CD30 positivity [48,78]. The majority of cases express TCRαβ (46–64%) with monoclonal TCR rearrangements. Other cases are TCR-silent or TCRγδ, and some cases show an NK-cell phenotype [78,79]. Recent GEP and cytogenetic studies have shown a strong upregulation of PD-L1 and T-cell related genes, a downregulation of CD56, and a loss of 14q11.2 (TCRA loci) in EBV-positive nodal TNKL—contrary to ENKTL, which suggests distinct entities [80].

## 9. Differential Diagnoses

Overall, an EBV-positive T/NK LPD diagnosis requires the integration of the clinical context (i.e., age, ethnic background, genetic predisposition, absence of underlying immunodeficiency, clinical presentation and course) together with immunomorphological features and EBV detection.

Differential diagnoses have been deeply discussed in a recent review by Hue et al. [81] and mostly include benign reactive or inflammatory conditions and other T-cell derived neoplasms, such as peripheral T-cell lymphoma NOS, T-cell large granular lymphocytic leukemia, hepatosplenic T-cell lymphoma, and adult T-cell leukemia/lymphoma. In most of these T-cell neoplasms, EBER in situ hybridization allows distinction from EBV-positive T/NK LPD. However, some peripheral T-cell lymphomas (such as angioimmunoblastic T-cell lymphomas (AITL)) contain variable amounts of EBV+ B-cells in their microenvironment. But unlike EBV-positive T/NK LPD, AITL tumor cells are EBV negative, and this disease is exceedingly rare in children. Moreover, specific mutations detected in AITL (such as RHOA^G17V^ and IDH2^R172^ mutations) have not been reported in EBV-positive T/NK LPD. Rare cases of aggressive mycosis fungoid (MF) and Sezary syndrome (SS) have been reported to be associated with EBV DNA detection [82,83,84]. However, these data rely on few studies, and further investigations are needed to clarify the ethiopathogenic role of EBV during these severe forms of MF or SS. Less commonly, aberrant expression of T-cell markers in EBV+ B-cell proliferations (such as plasmablastic lymphomas, EBV-positive diffuse large B-cell lymphomas, and extracavitary primary effusion lymphomas (PEL)) have been reported and may represent a diagnostic pitfall with EBV-positive T/NK LPD [85,86,87,88,89]. In these cases, the detection of clonal *IGH* gene rearrangements confirms the B-cell nature of the proliferation. In addition, PEL neoplastic cells not only exhibit EBV positivity but also show HHV8-associated latent protein LANA1 expression.

## 10. Conclusions

EBV-positive T/NK LPD are a group of uncommon disorders that can range from indolent reactive lesions to malignant and aggressive diseases, characterized by polyclonal or monoclonal proliferation of T- or NK-cells triggered by EBV infection, mostly of type II latency (Table 1).

EBV-positive T/NK LPD include various clinical forms that range from pauci or asymptomatic ones to fulminant progression. Among them, ENKTL and EBV-positive ANKL show the poorest outcomes, whereas EBV-positive LPD of childhood, including EBV-HLH and CAEBV (systemic and cutaneous forms), show more indolent courses. Importantly, T-cell clonality, the infiltrate density, and the proportions of EBV-infected cells in EBV-positive LPD of childhood predict neither outcomes nor potential evolution into more aggressive diseases such as ENKTL or ANKL. However, quantification of EBV DNA loads in peripheral blood is not only useful for diagnosis but also helpful for both disease monitoring and prognosis [21].

Not much is known about the pathogenesis and molecular features of EBV-positive T/NK LPD because of their rarity. The geographic and racial distribution pattern suggests that genetic predisposition related to a defective cellular immune response to EBV may contribute to an increased risk of developing EBV-positive T/NK LPD. Moreover, some genetic alterations (mostly involving JAK/STAT pathways, *DDX3X*, and *TP53*) have been reported, mainly in aggressive EBV-positive T/NK LPD. Nevertheless, these abnormalities remain insufficient to discriminate the different entities, making the diagnosis challenging. Thus, once an EBV-related LPD is established, a correlation between clinical data (i.e., age of patient, ethnic background, genetic predisposition, clinical presentation and course), and biological and histological features (especially the cell of origin) are required to achieve an accurate classification. The latter may be improved in the near future as a result of molecular techniques and a better characterization of EBV-positive LPD pathogenesis.

## Figures and Tables

**Table 1:** Classification and characteristics of EBV-positive T- and NK-cells lymphoproliferative disorders.

Disease Entity	Age Group	Phenotype	Histology	Molecular Findings
EBV-HLH	Pediatrics, adolescents	T CD8+ > NK	Lymphohistiocytic infiltrates Hemophagocytosis EBV+ cells with slight or no atypia	+/− Predisposing genetic conditions: mutations in *PRF1*, *UNC13D*, *STX11*, *STXBP2*, *SH2D1A*, *XIAP/BIRC4* genes.
CAEBV, systemic form	Pediatrics, adolescents	T CD4+ > CD8+ > NK	Non-destructive inflammatory lesions Scattered small-sized EBV+ cells	+/− Predisposing genetic conditions: HLA 26 and 52 loci [23]. Chromosomal abnormalities noted in few cases [3]. Alterations in NF-κB and JAK/STAT pathways [25]. Recurrent mutations involving *DDX3X*, *KMT2D*, *BCOR/BCORL1*, *KDM6A* and *TET2* genes [26]. Intragenic deletions in EBV genome [29].
CAEBV, cutaneous forms: -HV-LPD	Pediatrics, adolescents	T CD8+	Non-specific inflammatory changes Small or medium-sized EBV+ cells	Mutations in *STAT3*, *IKBKB*, *ELF3*, *CHD7* and *KMT2D* genes reported in one study [40].
-SMBA	Pediatrics, adolescents	NK	Polymorphic infiltrate Extensive necrosis, angiodestruction Large atypical EBV+ cells	Chromosomal abnormalities noted in rare cases [3].
STCLC	Pediatrics, adolescents	T CD8+ (primary) T CD4+ (post CAEBV)	Proliferation of bland EBV+ cells +/− Admixed atypical cells	Chromosomal abnormalities associated with poor outcomes [44]. Recurrent mutations involving *KMT2D*, *MFHAS1*, *STAT3*, *EP300*, *ITPKB*, *DDX3X NOTCH1* and *NOTCH2* genes [45].
ENKTL	Adults	NK	Proliferation of EBV+ atypical cells Angiocentricity, angiodestruction Necrosis	Cytogenetic aberrations: 6q16–27 and 17p15–22 deletions; 1q21–q44, 2q and 7q gains [62]. Mutations in *TP53*, *FAS*, *BCOR*, *STAT3*, *STAT5B*, *JAK3*, *DDX3X* genes. Alterations in epigenetic regulators (*KMT2D*, *KMT2C*, *ASXL3*, *ARID1A* and *EP300*) [27,61,66,67,68,69,70]. Gene expression: cell cycle/apoptosis, NFKB, NOTCH and JAK/STAT pathways deregulations, *MYC*, *RUNX3* and *EZH2* overexpression [63,64,65].
ANKL	Adults	NK	Patchy or diffuse destructive monotonous EBV+ infiltrates +/− Necrosis, hemophagocytosis	Cytogenetic aberrations: 1q23.1–24.2 and 1q31.3 gains; 7p15.1–p22.3 and 17p13.1 losses [62]. Recurrent mutations involving *STAT3* and *STAT5B* genes, RAS-MAPK pathways, *TP53*, *DDX3X*, *BCOR*, *TET2*, *CREBBP* and *MLL2* [45,74,75].
Nodal TNKL *	Adults, the elderly	T CD8+ > γδ or NK	Monomorphic or pleomorphic EBV+ cells proliferation +/− Necrosis, angiodestruction	Loss of 14q11.2 (TCRA loci) Gene expression: upregulation of PD-L1 and T-cell related genes (*CD2*, *CD8*, *CD3G*, *CD3D*, *TRAC*, *LEF1*); downregulation of *CD56* [78].

Abbreviations: EBV-HLH, EBV-positive hemophagocytic lymphohistiocytosis; CAEBV, chronic active EBV infection of T- and NK-cell type; HV-LPD, hydroavacciniforme-like lymphoproliferative disorder; SMBA, severe mosquito bite allergy; STCLC, systemic EBV-positive T-cell lymphoma of childhood; ENKTL, extranodal NK/T-cell lymphoma nasal type; ANKL, aggressive NK-cell leukemia; Nodal TNKL, primary EBV-positive nodal T-cell or NK-cell lymphoma. * Considered as a provisional entity.
